# (S)Pinning down protein interactions by NMR

**DOI:** 10.1002/pro.3105

**Published:** 2017-02-14

**Authors:** Kaare Teilum, Micha Ben Achim Kunze, Simon Erlendsson, Birthe B. Kragelund

**Affiliations:** ^1^ Structural Biology and NMR LaboratoryThe Linderstrøm‐Lang Centre for Protein Science, Department of Biology, University of CopenhagenOle Maaløes Vej 5, DK‐2200Copenhagen NDenmark

**Keywords:** affinity, dissociation constant, spectroscopy, ligand, chemical shift, binding, exchange

## Abstract

Protein molecules are highly diverse communication platforms and their interaction repertoire stretches from atoms over small molecules such as sugars and lipids to macromolecules. An important route to understanding molecular communication is to quantitatively describe their interactions. These types of analyses determine the amounts and proportions of individual constituents that participate in a reaction as well as their rates of reactions and their thermodynamics. Although many different methods are available, there is currently no single method able to quantitatively capture and describe all types of protein reactions, which can span orders of magnitudes in affinities, reaction rates, and lifetimes of states. As the more versatile technique, solution NMR spectroscopy offers a remarkable catalogue of methods that can be successfully applied to the quantitative as well as qualitative descriptions of protein interactions. In this review we provide an easy‐access approach to NMR for the non‐NMR specialist and describe how and when solution state NMR spectroscopy is the method of choice for addressing protein ligand interaction. We describe very briefly the theoretical background and illustrate simple protein–ligand interactions as well as typical strategies for measuring binding constants using NMR spectroscopy. Finally, this review provides examples of caveats of the method as well as the options to improve the outcome of an NMR analysis of a protein interaction reaction.

## INTRODUCTION

Proteins are intricate molecules and the many, diverse critical biological functions they have are intimately linked to their ability to bind and respond to various types of other molecules collectively here named ligands. Such ligands are miscellaneous and can be as small as a photon, an electron, or a hydrogen atom or they can be even larger than the protein itself in the form of another protein, DNA, RNA, or even a large, mixed complex. The response elicited by binding occurs through conformational changes of various amplitudes and can affect other ligand binding sites distant to the first through an allosteric process.^1^ Thus, the malleability of a protein scaffold can affect the responsiveness to ligands positively or negatively^2^, ^3^ in such a way that the protein may tightly control and regulate important cellular responses.

The strength by which a protein interacts with a ligand varies over many orders of magnitudes.^4^ However, the strength of an interaction, and hence the affinity between the two molecules is not proportional to the biological importance of a complex or to the specificity of the interaction, which is defined as the ratio of interaction energies.^5^ Thus, specificity towards a single ligand even exists for low affinity interactions and the formation of rather weak complexes can have important and critical biological consequences.^6^, ^7^ Similarly, high‐affinity complexes may be of lower specificity in the way that one protein may bind several different ligands with equally high affinity.^8^ Such promiscuous binding is prevalent for intrinsically disordered proteins.^9^ Regardless of these considerations, nonspecific interactions are often of very low affinity.

Since affinity is defined as the concentration of free ligand where half the protein population exists in a complex with the ligand (see Box 1), it becomes methodologically challenging to quantitatively describe a protein–ligand reaction when this is either ultra‐strong (*K*_d_ < nM) because quantification at low concentration set demands on method sensitivity, or when this is ultra‐weak (K_d_ > μ*M*), since quantification at high concentration set demands on solubility, availability, and sensitivity. The challenge emerges because the dynamic ranges of most optical and biophysical methods are in the μ*M*–n*M* concentration interval, resulting in a decrease of sensitivity at lower concentrations and signal saturation at higher concentrations. Compared to most other methods, Nuclear Magnetic Resonance (NMR) spectroscopy stands out as the more versatile, since it is capable of providing quantitative information for protein–ligand interaction with affinities lower than μ*M*, even when these are ultra‐weak.^10^, ^11^ Since NMR exploits the magnetic properties of the nuclei and thus measures properties of individual nuclei in a molecule, the method is also capable of separating signals from individual populations of molecules. Moreover, and importantly, the many diverse methods encountered by NMR spectroscopy makes it highly suitable for quantification of protein–ligand interactions, even at high concentrations.

In this review, we focus on how solution state NMR spectroscopy can be used to quantitatively and qualitatively describe simple protein–ligand interactions and we highlight where only qualitative information can be gained. We emphasize how the properties of a binding reaction such as the binding kinetics, the affinity of the complex, and the concentrations of the reactants, influence how the resulting NMR spectra appear, and we devise solutions to how these properties may be exploited best to improve the outcome of the analyses. The aim of the review is to provide the non‐NMR specialist with insights into the analyses and with tools to evaluate a protein–ligand interaction that has been studied by NMR. We do not intend to go into the more extended NMR theory underlying the study of protein complexes, or how NMR is used for drug discovery, and we will not discuss solid‐state NMR spectroscopy. Instead we refer to the many excellent reviews available on these topics.^12^, ^13^, ^14^, ^15^, ^16^

### The complex landscape of protein interactions

The reaction of a protein with a ligand cannot only be described by a simple equilibrium between the free protein, the free ligand and the complex. Rather the reaction is much more complicated and multidimensional energy landscapes are needed to describe the events from the unbound states of the molecules to the final complex.^17^, ^18^ Via diffusion, the protein and the ligand will occasionally encounter each other and depending on the precise orientations of the two molecules in this so‐called *encounter complex*, the complex will either be productive and proceed along the reaction coordinate or nonproductive leading to the subsequent dissociation of the complex.^19^, ^20^ Subsequent to the formation of a high‐energy, productive encounter complex, that is the *aligned encounter complex*, the two molecules will need to change conformation to adapt their surfaces either through a *conformer selection* process^21^, ^22^, ^23^ or through *induced fit* 24, 25 or both,^26^, ^27^ to optimize the final complementarity of the interacting molecules. However, most of these steps are not observable at equilibrium or in simple kinetic experiments, as the populations of the intermediates are extremely low and their lifetimes short. Thus, in most quantitative descriptions of protein–ligand reactions the approximation to the more simple is suitable.

**Figure 1:**
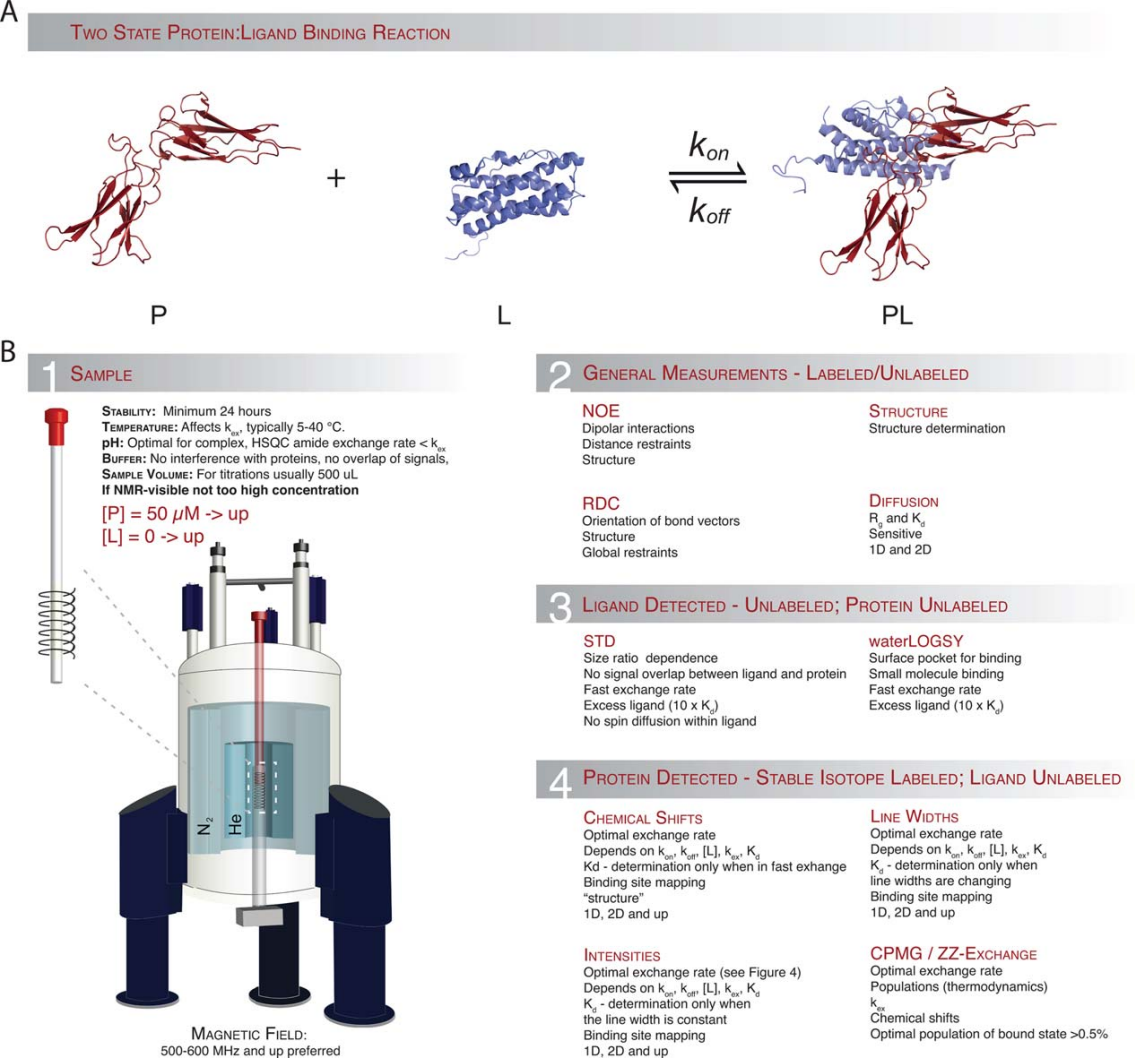
Protein–ligand interactions by NMR spectroscopy. **A)** Representation of a simple two‐state binding process. **B.1)** General considerations and limitations for NMR sample preparation. **B.2)** Experiments providing mainly structural information. **B.3)** Experiments useful for extracting binding information from non‐isotope labelled NMR samples, mainly used for drug discovery. **B.4)** Information that can be extracted from samples where either the protein or the ligand is labelled with stable isotopes.

We will initially describe why and how NMR is suitable for ligand binding investigations, explain the simple situations, which to a first approximation may account for most cases, and illustrate how and when different types of NMR experiments are applicable and list which type of information that can be extracted from each of these (Fig. 1). We will then demonstrate how knowledge of the reaction kinetics and the affinity of the interaction can be used to optimize the information that can be obtained from an NMR analysis of primarily isotopically labelled proteins. At the end, we will exemplify a number of complexes studied by NMR to illustrate how a seemingly simple reaction can have different and variable NMR spectral properties.

### Why use NMR to study protein–ligand interactions?

Most biophysical and structural techniques to study protein–ligand interactions fall in two groups: (1) techniques that measure the thermodynamics and/or kinetics of the interaction or (2) techniques that elucidate the structure of the interaction. The first set includes techniques such as isothermal titration calorimetry (ITC)^28^ and surface plasmon resonance (SPR)^29^, ^30^ to study binding thermodynamics and kinetics of protein–ligand interactions in bulk. This set also includes techniques such as dynamic light scattering (DLS),^31^ which is capable of measuring dissociation constants in bulk while also measuring the hydrodynamic radius, that is delivering very low‐resolution structural data. Lastly, analytical ultra‐centrifugation is also widely used to measure dissociation constants.^32^ A prime example of the second set of techniques is X‐ray crystallography. In many cases it can provide a static three‐dimensional picture of a protein and of protein–ligand complexes. Other techniques include electron paramagnetic resonance (EPR),^33^, ^34^ small‐angle X‐ray scattering (SAXS)^35^ or cryo‐EM,^36^, ^37^ and Förster resonance energy transfer (FRET),^38^ which in combination with suitable labels can deliver information (e.g. distance information) and thereby detect binding and deliver medium to low resolution structural data. Of course, fluorescent labels can also be used to acquire quantitative information about binding kinetics. While the above mentioned methods are not at all a comprehensive list of tools to investigate protein–ligand interactions it exemplifies that most techniques are specialized or can only deliver information for one or the other aspect, namely kinetics or structure.

NMR spectroscopy falls equally well in both groups since it can deliver structural details [Fig. 1(B.2)] at atomic resolution as well as quantitative kinetic measurements [Fig. 1(B.2–4)]. Although NMR spectroscopy may not be as straightforward to apply or to interpret as some of the other methods mentioned above one can use NMR to study complexes that exhibit ultra‐weak (mM) up to ultra‐strong (pM) binding. Since the outcome of the analyses depends strongly on the concentrations of protein and ligand, the kinetics and the differences in chemical shifts (see below), there are limitations to the type of information one may extract. Thus, some considerations should be taken into account before entering into either conducting or analyying a protein–ligand interaction by NMR spectroscopy. However, if nothing is known about the reaction prior to such endeavour, a trial‐and‐error analysis is often needed. In Figure 1(B) we have collected some of the most important points to consider before you conduct an NMR experiment including the general experimental parameters such as protein stability, protein concentration, buffer conditions, pH, and temperature [Fig. 1(B.1)]. The figure also highlights which kind of information that can be extracted from each type of NMR experiment, what obstacles are present, and which demands there may be at play.

### Using NMR to describe protein–ligand interactions

In NMR, a process in which a molecule of interest is exposed to a changing environment that originates from it populating two or more states is commonly referred to as *chemical exchange*. In the simplest case, an NMR experiment that investigates a chemical exchange in a sample containing a mixture of a protein and a ligand will display information about three different species, the free protein P, the free ligand L, and the complex PL. The three species are related by a simple two‐state reaction, where *k*_on_ and *k*_off_ are rate constants for the forward and reverse reactions, respectively [Fig. 1(A), Box 1].

**Figure 2:**
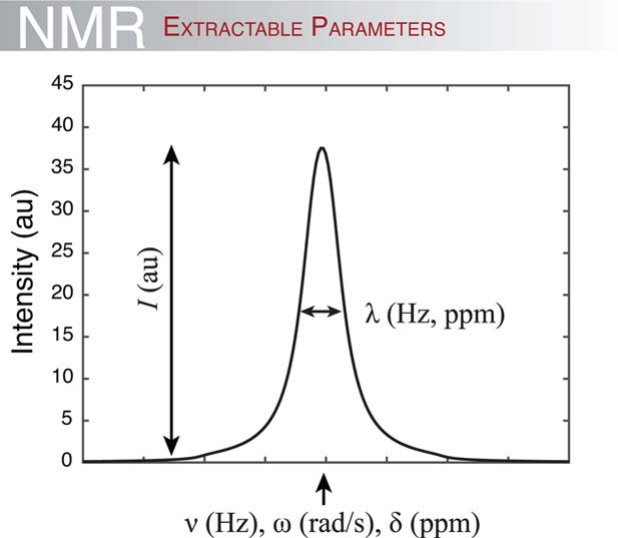
Extractable information from a 1D NMR signal. The intensity of the signal (or peak), *I*, depends on the concentration of the molecule (i.e. the resonating nucleus at the particular frequency) and the sensitivity of the spectrometer. The position of the peak is measured either in Hz, rad/s or ppm and provides information on the local chemical environment. A change in peak position thus reports on a change in the local environment. Finally, the width of the peak at half height, λ, typically measured in Hz or ppm gives information on the relaxation properties of the nuclei in question, and thus on the size and/or dynamics of the protein or the complex.

An NMR spectrum can in principle provide three different kinds of information on all three species (Fig. 2). Each NMR signal, termed a peak, is thus characterized by: (I) *its position in the spectrum*. This is reported either as the angular resonance frequency ω or the chemical shifts *δ* of the nuclei giving rise to the signal. Its position depends on the local, chemical environment, and any shift in the position of the peak can be used directly as a measure of changes in the chemical environment of the nucleus (e.g. when a ligand binds or the protein changes its conformation). (II) *The intensity of the peak*, which reports on the relative concentration and thus the population size of the nucleus resonating at that given frequency. (III) *The line width λ of the NMR peak*, which depends on the relaxation properties of the nucleus. *λ* is inversely correlated to the transverse relaxation time, *T*_2_, which again depends on the overall correlation (tumbling) time of the molecule and on possible exchange between different states.^39^

#### Methods for measuring binding when the ligand is small

For protein–ligand interactions, where the ligand is a small molecule or a peptide (<10 aa), binding can be monitored using either signals from the ligand, the protein or both and can bring information about which nuclei in the ligand are involved in binding and can determine binding affinities. Furthermore, observing the ligand circumvents the necessity of isotopic labelling (see below), which can be an advantage. Conversely, observing the ligand does not provide any structural information on the binding site on the protein. For estimating dissociation constants and to map interaction sites on the ligand, ligand‐detected NMR experiments such as saturation transfer difference (STD)‐NMR^40^ or Water‐Ligand Observed via Gradient SpectroscopY (water‐LOGSY)^41^, ^42^ may be useful [Fig. 1(B.3)]. For these experiments the protein must be much larger than the ligand, and the ligand must be in very large excess (typically 100:1). Before we delve into the general descriptions of protein–ligand interactions, we will briefly describe these two experiments.

During a binding event, protons on the ligand will come into proximity (<5Å) with the protein. Thus, if the protein is selectively saturated by an NMR radio frequency pulse, that is when we only aim for measuring the protein (see *glossary*), the protons of the ligand that are in contact with the protein will also become saturated and the intensity of their NMR peaks will be lowered, which is the basic principle of *STD‐NMR*. If the ligand at the same time is exchanging with many other ligands during the course of the experiment, which is often the case for small ligands, many ligand molecules will be affected during the saturation period. By subtracting an NMR spectrum containing information about saturation transfer from protein to ligand has been achieved from a spectrum without protein present, only those protons engaged in binding to the protein will show a signal in the resulting STD‐NMR spectrum. In this way, the binding epitope of the ligand is mapped. STD‐NMR has been widely applied for detecting binding of sugars, drugs, or synthetic molecules because their resonance frequencies are generally not overlapping with protein resonance frequencies.^43^, ^44^ However, epitope mapping of peptides has also been reported.^45^

In the *Water‐LOGSY* experiment, similar selective saturation is applied but involving bulk water protons and exploiting *the NOE effect*,^46^, ^47^ involving dipole–dipole interactions. Here bulk water is saturated and either via bound water in the interface or exchange of labile protons in the binding interface with water, or water molecule in the protein–ligand surface, the signals from the ligand in contact with the protein is mapped. In this case, this results in positive signals for interacting protons, and negative signals for noninteracting protons. Several excellent reviews discuss these ligand observing techniques in much more detail.^12^, ^48^, ^49^

#### Protein observed methods for measuring binding events

In contrast to the ligand observed methods, labelling the protein with stable isotopes, typically 15N, 13C, or both, can provide residue‐specific, structural information about the binding interface, and give a reasonable estimate of binding constants in both fast and slow exchange regimes (see below) [Fig. 1(B.4)]. Moreover, isotopic labelling also allows for the possibility to select NMR signal from the binding partner that gives the best observables in terms of peak resolution in the NMR spectra. Chemical shifts as observed for the various species are powerful probes of binding interactions. However, the appearances of the individual peaks, in terms of the intensities and line widths, can vary and quantification of the actual binding event depends on several properties, as will be described below.

A protein in a simple two‐state reaction [Fig. 1(A)] will give rise to two species with different resonance frequencies, $\omega_{P}$ and $\omega_{PL},$ and a resonance frequency difference of $\Delta \omega =\omega_{P}-\omega_{PL}$. However, and importantly, the appearance of the different species of the protein in the NMR spectra varies and depends strongly on the concentrations of the species, the dissociation constant, *K*_d_, and the *exchange rate of the reaction*, *k*_ex_:

(5) $$k_{\text{ex}}=k_{\text{on}}\left[L\right]+k_{\text{off}}$$

The rate constants also determine the populations of the free protein, P, and bound protein, PL, which at equilibrium is given by

(6) $$p_{P}=\frac{k_{off}}{k_{on}[L]+k_{off}},\;p_{PL}=\frac{k_{on}[L]}{k_{on}[L]+k_{off}}$$

**Figure 3:**
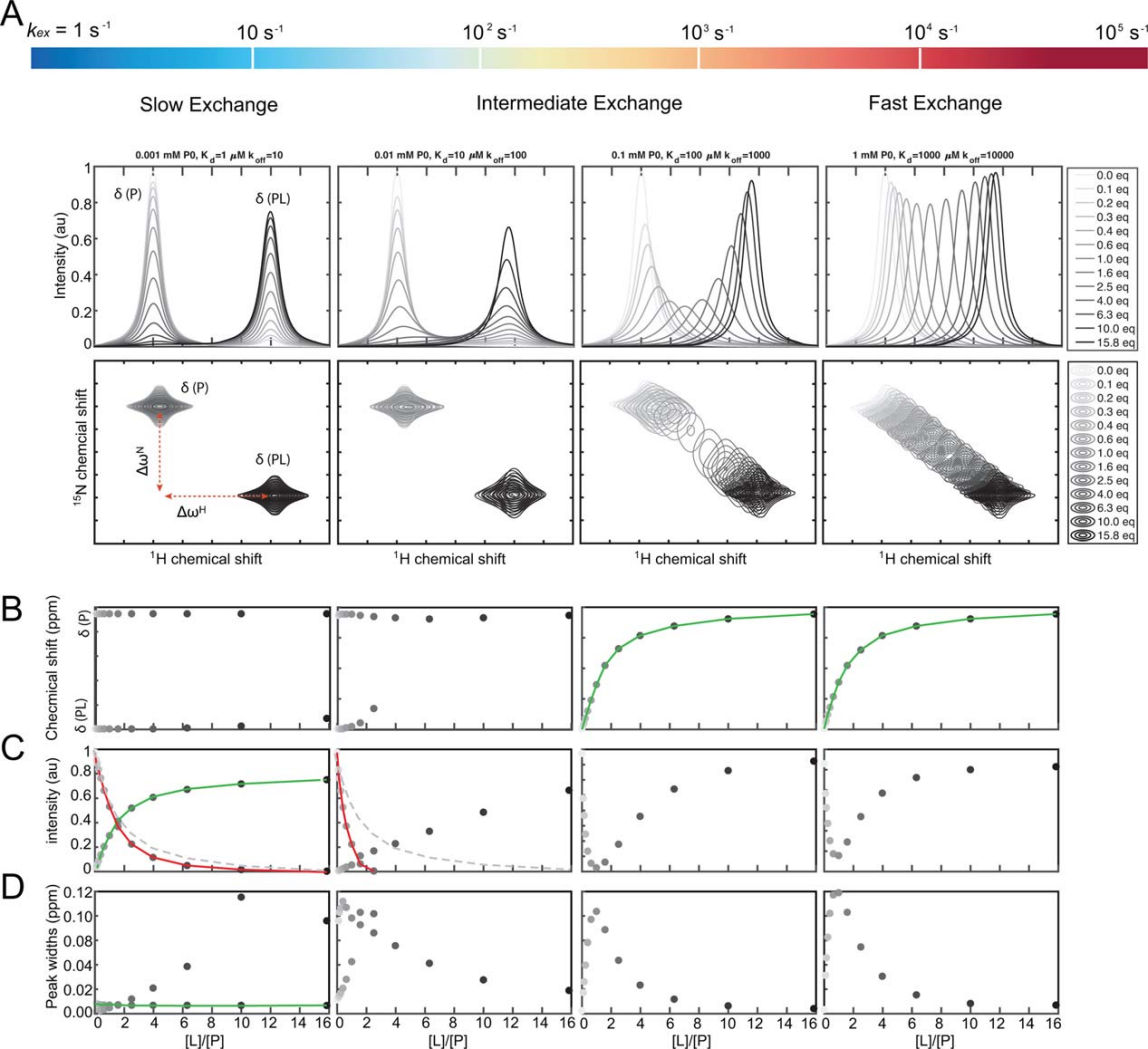
NMR quantification of binding events occurring in different exchange regimes. **A**) 1D (top) and 2D (bottom) spectra of a protein titrated with a ligand ranging from 0 (light grey) to 15.8 (black) molar equivalents of ligand compared to the initial free protein concentration. Changing the exchange rate (*k_ex_*) from slow to fast (left to right) compared to the difference in resonance between the free and bound states, Δω, changes the appearance of the spectra. In the slow exchange regime (left), the quantification will rely on either the peak intensities of the unbound protein or of the protein–ligand complex. In the fast exchange regime (right), the chemical shift of the observed peak will be a weighted average of the population of the two states, and thus, the binding constant can be readily determined from the chemical shift. In this case, quantification is feasible in both dimensions, and is often expressed as a normalized Euclidian distance between the different states (see text). **B‐D)** The extractable parameters obtainable from the spectra during the ligand titration: (**B**) The chemical shift in ppm or Hz; (**C**) the normalized intensity of the NMR peak in arbitrary units, and (**D**) the NMR peak width at half height measured in ppm or Hz. The solid green lines indicate when reliable binding constants can be extracted from fitting the observed parameter, the solid red lines indicate false estimates of the binding constant and the grey dashed lines show the correct calculated binding curve for the red lines.

Chemical exchange is typically divided into three categories (*slow*, *intermediate,* and *fast exchange*) based on the magnitude of *k*_ex_ (Box 2) relative to the size of the difference in resonance frequency (or chemical shift) of a given nucleus in P and PL (Fig. 3). Moreover *k*_ex_ must be compared to the resonance frequencies measured in *angular frequency,* Δ*ω*. We will now in more detail describe the three different exchange regimes.

When the exchange rate is much slower than the observed difference in resonance frequency ${(k}_{ex}\ll \left|\Delta \omega \right|)$, the exchange is said to be *slow on the NMR time scale* (Box 2), and each state and the corresponding resonance frequencies can be individually observed [Fig. 3(A), left]. For a set of resonances in slow exchange the peaks do not move in the spectrum when their relative population changes. Consequently, the peak positions will not provide a quantitative measure of the binding event, as illustrated in Figure 3(B), left. However, the relative population sizes of the states can be determined from the peak intensities, and the titration of a protein–ligand binding process in slow exchange can be followed directly by the changes in intensities of the bound state [Fig. 3(C), left].

At *the intermediate time scale* ${(k}_{ex}\approx \left|\Delta \omega \right|)$, also referred to as *coalescence*, only a single peak, which is severely *broadened* by the exchange process, is observed. The intensities and positions of peaks from nuclei in intermediate exchange are highly uncertain and the quantitative interpretation of this situation is fairly complicated [Fig. 3(A,B), middle], and often not feasible. Importantly, it is not possible to assign a given ligand binding process to one of the three exchange regimes without knowing all details of the experimental setup. This includes the operation frequency of the NMR spectrometer, the temperature, the concentration of the free ligand, and the *on*‐ and *off*‐rates all influence the appearance of the NMR spectra. This will be elaborated on below.

Finally, when the exchange rate is *fast on the NMR time scale* ${(k}_{ex}\gg \left|\Delta \omega \right|),$ only a single peak will be observable at the population‐weighted average position of their respective resonance frequencies given by $\omega_{Obs}=p_{P}\omega_{P}+p_{PL}\omega_{PL}$ where *p*_P_ and *p*_PL_ are the populations of the free and bound protein, respectively. In this case, a binding event can be observed from plotting the peak position as a function of the concentration of added ligand, which will provide a sigmoidal log‐transition from the free to the bound state [Fig. 3(B), right]. In contrast, plotting the intensities of the peak will not correctly provide a quantitative measure of the binding as illustrated in Figure 3(C), right.

As demonstrated in Figure 3 it appears less straightforward to quantify binding constants (*K*_d_ values) using the position given by the chemical shift or intensity of an NMR signal, when outside the fast exchange regime. Even for a binding event involving only two components without any intermediates (two‐state binding) the choice of method depends largely on the system at hand where the labelling strategy, the relative concentration and/or the desirable observable must be changed from system to system. For this reason, it is often an advantage to have an idea of the binding strength of the interaction, as well as the general stability of the individual components. However, with such knowledge one may be able to tune the concentrations and condition to extract the needed information.

### Quantification of binding using multidimensional NMR

Modern biomolecular NMR relies largely on multidimensional spectra where magnetization is transferred between two or more nuclei. This allows for observation of residue specific chemical shifts in even very large proteins.^50^, ^51^, ^52^, ^53^, ^54^ The most widely used heteronuclear experiment in solution state NMR is the ^1^H‐^15^N *HSQC* spectrum that serves as a protein fingerprint where correlations of the amide proton and amide nitrogen are measured [Fig. 3(A), bottom]. In the ^1^H‐^15^N HSQC spectrum the magnetization starts on the amide protons, and is then transferred to the amide nitrogen where it develops and finally transferred back for acquisition on the protons. In this way, each peak in a ^1^H‐^15^N HSQC spectrum is labelled with the chemical shifts of the amide proton (direct dimension) and the covalently attached nitrogen (indirect dimension).

In the fast exchange regime, the chemical shift in either of the two dimensions, or the combined shifts, Δ*δ*_comb_, (the weighted *Euclidian distances*) can be used for visualizing and fitting of a binding curve.^55^, ^56^, ^57^, ^58^ As an example, a binding event causes chemical shift changes of 0.7 and 1.2 ppm in the proton and the nitrogen dimensions, respectively, in a ^1^H‐^15^N HSQC. A weighting factor balances the chemical shifts in the proton and nitrogen dimensions and is often set to $\left|\gamma_{N}\right|/\left|\gamma_{H}\right|=27.1/267.5∼0.$1, where *γ* is a constant existing for each type of nuclei termed the gyromagnetic ratio. Therefore:

(7) $$\Delta \delta_{comb}=\sqrt{({\Delta \delta H)}^{2}+({0.1\cdot \Delta \delta N)}^{2}}=\sqrt{({0.7)}^{2}+({0.1\cdot 1.2)}^{2}}=0.710\;ppm$$

When residing in the slow exchange regime, the intensities measured in the direct dimension can also be used for monitoring the binding event. However, as the intensities are also susceptible to changes in dynamics of the bound *versus* unbound states as well as line broadening when approaching the intermediate exchange regime [Fig. 3(B), second column], one must be careful when using intensities for quantifying binding events. This also holds true for quantification of dynamics based on the line shapes. These depend also on the evolution of magnetization throughout the pulse sequence used, for example variable relaxation delays, effects from multiple coherence transfers during evolution or different relaxation properties of the free/bound state,^59^, ^60^ and thus are not easily interpretable.

There are other, more sophisticated NMR techniques that can be used to characterize and quantify binding processes in the intermediate to slow exchange regime, including Carr‐Purcell‐Meiboom‐Gill (CPMG) relaxation dispersion^61^, ^62^ and ZZ‐exchange.^63^ In brief, CPMG uses a train of equidistant 180° degree pulses to refocus transverse magnetization at a corresponding frequency *ν*_CPMG_ during a fixed relaxation delay. By observing the NMR signal intensity changes when varying *ν*_CPMG_ one can quantify exchange contributions to the relaxation rates that originate from processes on the micro‐ to millisecond timescale. This technique can be very effectively used to probe the *k*_ex_ and the population of interconverting conformational states of proteins (>0.5% populated minor state), where the minor state is often called *the excited state* or *the invisible (dark) state*.^64^ CPMG can also be used to probe complex formation and kinetics, when the population of the complex is low and therefore not visible in a normal HSQC type experiment. In favorable cases even the kinetics of an encounter complex formation can be followed using CPMG.^65^

ZZ‐exchange experiments can be used to probe even slower processes where *k*_ex_ range between 0.1 and 10 s ^−^ 1. In these experiments longitudinal magnetization is created, for example on isotopically labelled amide nitrogen atoms and allowed to transfer from one state (typically the major/unbound state) to the other state (typically the minor state/bound state) during a mixing time. In many cases, the population and *k*_ex_ of the states can be determined from the volume of the cross‐peaks at different mixing times_._ While this technique can be used to study slow conformational exchange^63^ it has also been used to study slow binding processes of protein and DNA.^66^

### Reliable fitting of NMR observed binding events

As a good rule of thumb, a suitable estimate of the *K*_d_ can be obtained when the total concentration of protein, *P*_0_, is in the same range as (or less than) the *K*_d,_ and the total ligand concentration, *L*_0_ is titrated between 1/10 and 10 times the *K*_d_.^12^ Importantly, accurate determination of the *K*_d_ relies on reaching complete saturation. For a simple two‐state binding in fast exchange the *K*_d,_ can be fitted by the function:

(8) $$\Delta \delta_{obs}=\Delta \delta_{max}\frac{{\left([P\right]}_{0}+{\left[L\right]}_{0}+K_{d})-\sqrt{{{\left[P\right]}_{0}+{\left[L\right]}_{0}+K_{d})}^{2}-4{\left[P\right]}_{0}{\left[L\right]}_{0}}}{2{\left[P\right]}_{0}}$$

where $\Delta \delta_{obs}$ and $\Delta \delta_{max}$ are the observed/combined and maximum chemical shift changes, respectively.

As can be seen from Figure 3, fitting of intensities is not always a reliable route to quantitative data. Although the decay of the intensities follows a hyperbolic curve reminiscent of a binding curve, caution should be taken, as the relaxation properties of both states will affect the NMR peak intensities. This is demonstrated in Figure 3(C), where the decay of the free state and the build‐up of the bound state do not cross at 50% bound. The reason for this behavior is that the correlation time for the free protein, and hence the peak width is influenced by the tumbling in the bound state, which differs from the free protein if the size of the protein–ligand complex is significantly larger to that of the protein. Thus, fitting leads to an overestimation of *K*_d_, and binding appears stronger than it is. Instead, fitting the intensities of the bound state, where the line width stays constant, will result in a reliable *K*_d_ determination.

In the cases where the exchange rate is in the intermediate exchange regime, analysis purely based on chemical shifts or intensities will unfortunately not provide a reliable quantification of the binding strength of the complex, as the peaks are broadened and thus not reflecting the true populations. The same holds true if more than one transition/reaction occurs, which can result in complicated chemical shift or intensity changes, or if the dynamics of the complex is considerably altered as a result of the binding event. Of important value, methods for analyzing line shapes of one‐ and multidimensional spectra have for these cases recently been presented in excellent work^59^, ^60^, ^61^, ^67^ and will be useful for the extraction of quantitative measures of the binding reaction.

### The NMR timescale and relations to the binding kinetics

As noted above, it is necessary to have an estimate of the kinetic parameters, the ligand concentration and differences in chemical shifts between the resonances from a protein in its free and ligand bound states before its behavior in NMR can be predicted and the type of NMR experiment most suitable selected. Conveniently, the NMR spectral appearance can be simulated and the influence of the different parameters thereby easily illustrated. Here, we have simulated how the spectral appearance depends on *K*_d_, *k*_on_, *k*_off_ and [L], which provides an overview of when access to useful NMR data is possible.

**Figure 4:**
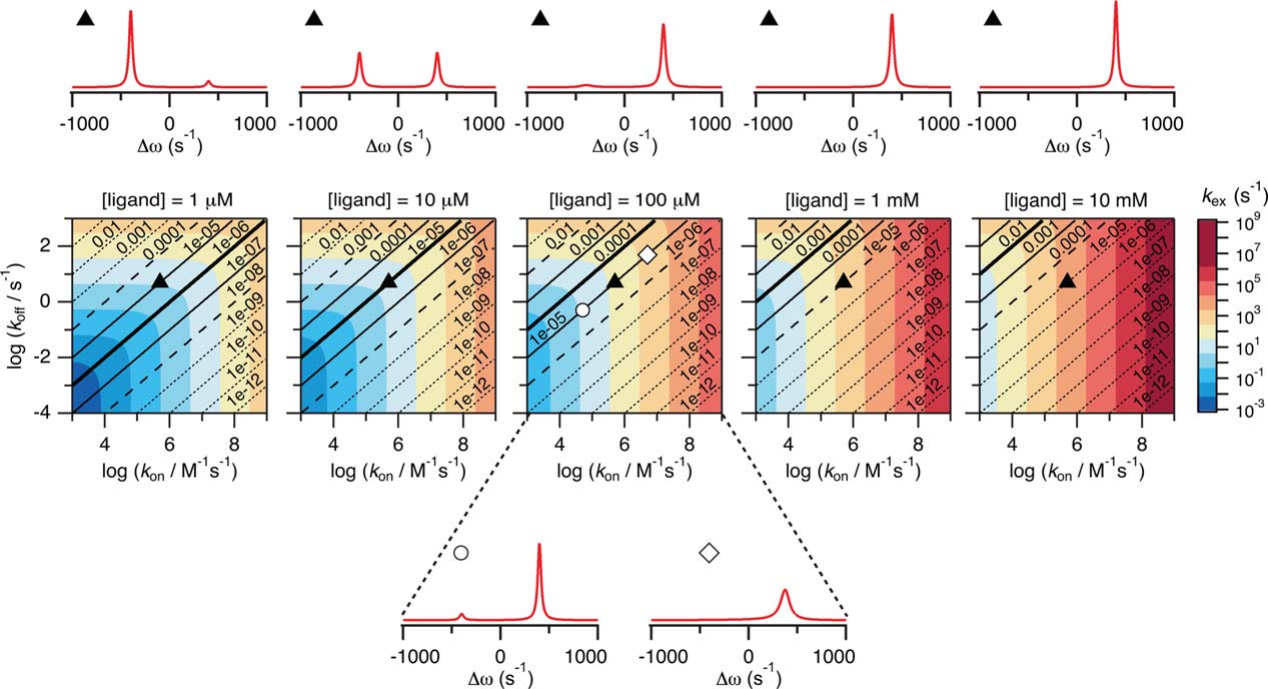
The relationship between binding kinetics and ligand concentration. The figure shows how the kinetics for a protein–ligand binding reaction changes with the concentration of the free ligand, which varies from 1 μ*M* (leftmost plot) to 10 m*M* (rightmost plot). The contour plots in the middle of the figure are coloured according to *k*_ex_ where *k*_on_ and *k*_off_ are varied in intervals typical for protein–ligand interactions. Blue regions are in slow exchange while orange/red regions are in fast exchange. The diagonal lines are isoaffinity lines showing regions in the plots with the same *K*_d_. The bold solid lines are where *K*_d_ is equal to [L], the light solid lines are where *K*_d_ is 10‐fold higher or lower than [L], and the dashed lines are where *K*_d_ is 100‐fold higher or lower than [L]. A simulated NMR spectrum is shown above each plot for a binding reaction with *k*_on_ = 5 × 10^5^ *M* ^−1^s^−1^ and *k*_off_ = 5 s^−1^ (corresponding to the black triangles in the contour plots). It is seen that the binding process changes from being in slow exchange at the low ligand concentration to being in fast exchange at the high ligand concentration. Two additional simulated NMR spectra are shown below the central contour plot for [L] = 100 μ*M*. These spectra illustrate binding reactions with the same *K*_d_ as for the reaction simulated above the plots, but with rates 10‐fold lower (white circle; *k*_on_ = 5 × 10^4^ *M* ^−1^s^−1^ and *k*_off_ = 0.5 s^−1^) and rates 10‐fold higher (white square; *k*_on_ = 5 × 10^6^ *M* ^−1^s^−1^ and *k*_off_ = 50 s^−1^).

One effect that may occur during a titration is that a given binding reaction changes from being in slow exchange at low ligand concentrations to being in fast exchange at high ligand concentrations. The situation is illustrated in Figure 4 for a process with *k*_on_ = 5 × 10^5^ *M* ^−^ 1s ^−^ 1 and *k*_off_ = 5 s ^−^ 1. The five spectra at the top of Figure 4 show how the exchange regime changes from slow to fast during a titration with increasing amounts of ligand added. It should be noted, that the lower limit for any protein NMR experiment to be completed in a reasonable amount of time is currently around 50 μ*M* and it will be extremely difficult to prepare a sample of a protein–ligand complex at 50 μ*M* with a free ligand concentration of 1 μ*M*. This situation, which corresponds to the leftmost spectrum at the top of Figure 4 is only included to illustrate the principle.

It is important to have in mind that only *k*_on_, *k*_off_, and [L] determine the exchange rate. The dissociation constant, *K*_d_, on the other hand says nothing about whether a binding reaction will appear in slow, intermediate, or fast exchange. This is illustrated in the contour plots in Figure 4 where the *K*_d_ *isoaffinity lines* (the black lines, along which *K*_d_ is constant) all go through areas of slow (blue) and fast (red) exchange. The different behaviors possible for binding reactions with *K*_d_ of 10 ^−^ 5 *M* at a free ligand concentration of 100 μ*M* are illustrated by the three spectra above and below the middle contour plot where *k*_on_ and *k*_off_ changes in steps of a factor of 10. Still there is a tendency for strong binding reactions to be in slow exchange and weak binding reactions to be in fast exchange. The underlying reason for this is that a small *K*_d_ inevitably will result in low free ligand concentrations until the protein become saturated. Consequently, *k*_ex_ will be much lower for strong interactions than for very weak interactions (*K*_d_ > m*M*) where very high concentrations of free ligand are needed to populate the protein–ligand complex (the plots to the left in Figure 4 are more blue than those to the right).

Conclusively, one may alter the appearance of the NMR spectra by changing the concentration of the protein or the ligand, and this change can influence the possibility of extracting useful and quantitative information from the NMR data. Careful planning and knowledge on why the NMR spectra change, is thus important.

### Realistic protein–ligand interactions—it is (not) so simple!

Experimentally, we know now from Figures 3 and 4 that a binding experiment followed by NMR depends on many more parameters than just the affinity (*K*_d_). In the following we will present a selected set of quantitative as well as qualitative NMR analyses of protein–ligand interactions where the ligand is a peptide, a small protein or a large protein. Together these data cover examples in the three exchange regimes, they represent complexes with affinities spanning from m*M* to n*M*, and they can be evaluated in structural contexts, as the structures of the complexes are known. Furthermore, details of their study have been published and can thus be further explored.

**Figure 5:**
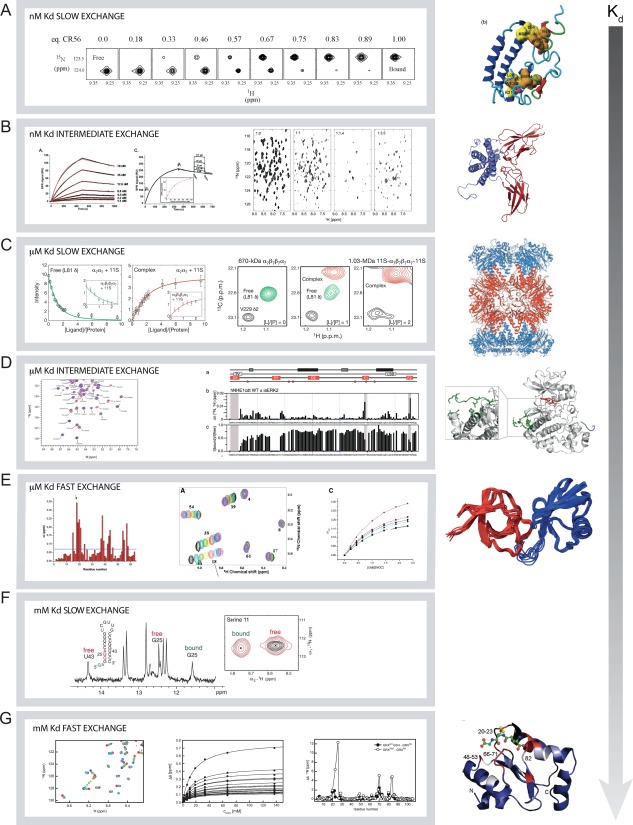
Examples of protein–ligand interactions studied by NMR spectroscopy. Seven different examples are shown with difference in exchange rates and binding affinities. A) Binding of CR56 to 15N‐RAP (PDB 2FYL) where the affinity is in the nM range and the process in slow exchange.^68^ The NMR spectra are extracts from 15N,^1^H‐HSQC spectra and the peak from the free state of Glu40 is disappearing and the peak from the bound state is appearing. Figure modified and reprinted from reference (68), copyright (2006), with permission from Elsevier. B) Binding of prolactin to the extracellular domain of the prolactin receptor measured by SPR^70^ (left) and by 15N,^1^H‐HSQC NMR spectra^69^ (right) (PDB 1RW5, 3NPZ). The spectra show the free prolactin (1:0) and with increasing molar amount of the extracellular domain of the prolactin receptor. Figures are reproduced from reference (70, left) and reference (69, right) with permission. Copyright (2007), The American Society for Biochemistry and Molecular Biology, and copyright (2005) Oxford University Press, respectively. C) Formation of the MDa complex between the 20S proteasome and the 11S activation domain which is in slow exchange and has μ*M* affinity.^54^ Peaks from both the free and the bound states of 13C‐methyl labelled 20S proteasome are observed in the methyl TROSY spectra. Reprinted by permission from Macmillan Publishers: Nature (reference 54), copyright (2007). D) Interaction between the intrinsically disordered C‐terminal tail of NHE1 with ERK2 where the binding process is in the intermediate exchange regime and with μ*M* affinity. Extract from the HSQC NMR spectra (left) where peaks disappear and mapping of change in peak intensities in these spectra as a function of residues shown as the ration between the free and bound states.^71^ Grey shaded areas indicate the three interaction sites. The three lines a) show the position of the transient helices in the disordered regions, and in red the possible kinase docking sites. Stars indicate potential ERK2 phosphorylation sites. Reproduced with permission from (71), copyright (2016) Springer Nature. E) Binding in the fast exchange regime with μ*M* affinity between the SH3 domain of CD2 associated protein (CD2‐AP) with ubiquitin.^72^ To the left is shown the combined chemical shift changes pr. residue, the middle show the individual spectra of the titration and c) the determination of *K*d from fits to the change in chemical shifts. Figure is reproduced from (72), copyright (2013) PLOS. F) Slow and weak binding of a GGA motif in the stem of an RNA hairpin to the protein RsmE from *P. fluorescens*. Peaks from both RNA (1D ^1^H‐spectra) and protein (2D ^1^H,^15^N‐HSQC spectra) in the free and bound states are observable^74^. Figure reproduced from (74) with permission. Copyright (2014), Oxford University Press. G) Interaction with glutathion with glutaredoxin is weak and the process is in fast exchange. The changes in chemical shifts in the ^1^H,^15^N‐HSQC NMR spectra can adequately be fitted to determine a dissociation constant^25^. Middle and right reproduced, with permission, from reference (25), copyright (2011) American Chemical Society.

For protein–ligand complexes with *K*_d_ in the n*M* range, NMR can be used to characterize the structure of the complex and map the binding sites, but not to determine the value of *K*_d_. A proper titration would require that samples with varying free ligand concentrations in the n*M* range could be prepared. However, with the sensitivity of NMR this is not feasible. As a first example, a double‐domain, CR56, from the lipoprotein receptor‐like protein (LRP) was titrated with the first domain of receptor‐associated protein (RAP), an ER‐resident chaperone. The complex is in slow exchange on the NMR timescale giving rise to peaks from the free state disappearing and peaks from the bound state appearing [Fig. 5(A)].^68^ As can be appreciated, there is no change in line widths, and hence the change in peak intensity at the different positions could in principle be used to extract *K*_d_. However, as the concentrations in the NMR experiment were well above *K*_d_, the titration was instead used to determine the stoichiometry, and *K*_d_, was measured to be 0.6 μ*M* by SPR.^68^ Also, the changes in chemical shifts were crucial for defining which residues were involved in binding; information that was used as input for modeling the complex.

Intermediate exchange is also possible in the n*M* range [Fig. 5(B)]. This is illustrated by the binding of the extracellular domain of the prolactin receptor to 15N‐labeled prolactin, first in a 1:1 molar ratio, which resulted in the disappearance of many signals. *K*_d_ for this site is 6 n*M* and *k*_on_ is 10^4^ s ^−^ 1 as determined by SPR (left).^69^, ^70^ Since prolactin binds two copies of the extracellular domain, and *K*_d_ for the second site is 33 μ*M*,^70^ further addition of the extracellular domain worsened the spectral outcome, and almost all signals from prolactin disappear because of exchange broadening [Fig. 5(B)].

For medium binding affinity where *K*_d_ is in the μ*M* range, NMR can monitor examples of the three exchange regions as illustrated amply in the literature. Here, we present three studies. In the first example, NMR spectroscopy was used to characterize binding of the 11S activation domain to the 20S proteasome.^54^ This work by Sprangers and Kay beautifully illustrates how even very large systems (up to 1 MDa), can be studied by NMR. Specific isotope labelling of side chain methyl groups combined with *TROSY* allowed quantification of the binding process on a sample of only 9 μ*M*! The binding of the activation domain is in slow exchange and fitting of the intensity changes on either the free 20S proteasome or the complex gave *K*_d_ = 12 μ*M* [Fig. 5(C)]. Another example of a complex with μ*M* affinity is the interaction between the intrinsically disordered tail of the sodium proton exchanger 1 (NHE1cdt) and the extracellular signal‐regulated kinase 2 (ERK2)^71^ [Fig. 5(D)]. In this complex, where *K*_d_ ≈ 30 μ*M*, NHE1cdt is in intermediate exchange between the free and the ligand bound states. In the spectrum it is clear that the peaks from Leu684 and Val686, which are part of a D‐domain in NHE1cdt, completely disappear. In this case, changes in intensities were used to guide mutagenesis, which pinpointed three contact sites in NHE1cdt for ERK2.

The last example is the interaction between the SH3 domain of CD2 associated protein (CD2‐AP) with ubiquitin, which is in fast‐exchange, has μ*M* affinity range, and where the peaks move as a population averaged chemical shift.^72^ The changes in chemical shift can be plotted as function of the ligand concentration to determine *K*_d_. Moreover, the data can be used to map the binding site on the SH3 domain (left) [Fig. 5(E)].

For weak protein–ligand complexes that bind with m*M* affinities, NMR spectroscopy is one of very few techniques that allow quantification of *K*_d_. As the ligand has to be present in m*M* concentrations to populate the protein–ligand complex the reaction most often is in either fast or intermediate exchange. However, there are for example RNA complexes with mM affinity where the kinetics gives rise to slow exchange. This behavior is observed for the binding of the drug theophylline to an RNA aptamer.^73^ In this rather peculiar case *K*_d_ = 7 m*M*, while *k*_on_ = 600 s ^−^ 1 *M* ^−^ 1 and *k*_off_ = 1.5 s ^−^ 1. The RNA binding protein RsmE from *Pseudomonas fluorescens* binds several different RNA hairpins with affinities ranging from n*M* to m*M*.^74^ For the two weakest binding hairpins, where the binding motifs are part of the stable secondary structure in the stems of the hairpins, the binding processes are in clear slow exchange as peaks from RNA and protein in both the free and bound states are observed in the NMR spectra [Fig. 5(F)]. Still, *K*_d_ for the interactions were only 0.3 and 2.7 m*M*, respectively.^74^

A weak binding reaction in fast exchange is seen in the enzyme glutaredoxin that catalyses the (de‐)‐glutathinoylation of Cys‐residues. Glutaredoxin becomes product inhibited by glutathione at m*M* concentration and quantification of this process was essential to interpret kinetic data on the enzyme catalysis [Fig. 5(G)]. By following the chemical shift changes *K*_d_ was determined to 15 m*M*.^25^ This situation is similar to the binding processes in fast exchange with higher affinity described above.

Collectively, the above experiments show that the appearance of the NMR spectra is not directly related to the affinity of the complex. Rather, it reflects the concentrations of the individual components, the kinetics of the interactions and hence the exchange rate. Although the NMR analyses in some cases did not provide any meaningful quantitative data, it still provided useful insight into the protein complex in terms of the structure of the complex and in mapping of the binding site. Thus, NMR spectroscopy applied to protein–ligand interactions is close to being a true win‐win approach to understanding protein function.

### Final remarks

Via simulated and real experiments, this review intends to explain and visualize how (1) a protein–ligand interaction can be quantitatively addressed by solution state NMR spectroscopy and (2) to what extent various parameters, such as the concentration of the interacting species, the kinetics of the interaction as well as affinity of the complex influence the appearance of the NMR spectra and the analyses. From the short overview of the practical considerations (Fig. 1), the examples and the brief theoretical explanation provided in the boxes, the reader should now be equipped with the possibility to suggest, analyze, and evaluate quantitative NMR analyses on protein–ligand interactions.

**Box 1**

**Binding equilibrium**

The simplest case of a protein binding to a ligand occurs when there is only one binding site for the ligand in the protein. Consider therefore a protein P that binds a ligand L.

(1) P+L⇋PL

At equilibrium when there are no net changes in the concentrations of the reactants and the product, the ratio between the concentrations of the molecules in the free state ([P] and [L]) and the concentration of the complex, [PL], is given by the equilibrium constant, *K*_eq_. For the reaction above, the equilibrium constant is called the association constant, *K*_a._The unit of *K*_a_ is M ^−^ 1 and often the equilibrium constant for the dissociation reaction is reported instead. This is the dissociation constant, *K*_d_ = 1/*K*_a_, and *K*_d_ is simply the free ligand concentration at which 50% the protein population is bound to the ligand.

(2) $$K_{d}\;=\;\frac{\left[P\right][L]}{\left[PL\right]}$$

**BOX 2**

**What is the “NMR time scale” and how does it relate to the exchange rate?**

The ‘NMR time scale’ term is typically subject to some confusion because it used in a variety of contexts. When probing a protein–ligand interaction using standard one‐dimensional ^1^H spectra or two‐dimensional ^1^H–15N HSQC spectra, the lifetimes of the unbound and bound states, τ, determine how accurately one can determine the resonance frequencies, $\omega_{P}$ and $\omega_{PL}$, respectively. The uncertainty in determining the resonance frequencies will be directly related to the lifetime^75^ by the following equation:

(3) $$\Delta \omega =\frac{\hbar }{\tau }$$

Here Δω is the difference in resonance frequencies between the sampled states (in rad/s), and ℏ is Planck's constant. Assuming only two states, then Δω is directly proportional to the energy difference (ΔE=Δω·ℏ) and the uncertainty principle now tells us that as long as *τ* is very long Δω can be measured accurately, i.e. both states give a signal each. However, if the lifetimes of the states are very short, the difference in frequency cannot be measured resulting in a collapse of measured signals, which is often called *coalescence*. More precisely, this happens when:

(4) $$k_{ex}\;(s^{-1})=\frac{1}{\tau }=\Delta \omega \;(rad/s)$$

This results in three different regimes that shape the appearances of the NMR spectra **(FIGURE** 3 **A)**:

- *Fast exchange* regime where *k*_ex_ ≫ |Δω|
- *Intermediate exchange* regime where *k*_ex_ ∼ |Δω|
- *Slow exchange* regime where *k*_ex_ ≪ |Δω|

For protons Δω typically ranges from 10 to 10,000 s ^−^ 1.
